# Pancreatic Cancer Cells Undergo Immunogenic Cell Death upon Exposure to Gas Plasma-Oxidized Ringers Lactate

**DOI:** 10.3390/cancers15010319

**Published:** 2023-01-03

**Authors:** Lea Miebach, Hager Mohamed, Kristian Wende, Vandana Miller, Sander Bekeschus

**Affiliations:** 1 Department of General, Thoraxic, Vascular, and Visceral Surgery, Greifswald University Medical Center, 17489 Greifswald, Germany; 2 ZIK *plasmatis*, Leibniz Institute for Plasma Science and Technology (INP), Felix-Hausdorff-Str. 2, 17489 Greifswald, Germany; 3 Emergex Vaccines Holding Limited, Doylestown, PA 18902, USA; 4 Department of Microbiology and Immunology, Institute for Molecular Medicine and Infectious Disease, Drexel University College of Medicine, Philadelphia, PA 19102, USA

**Keywords:** kINPen, oncology, peritoneal carcinomatosis, plasma-conditioned liquid, plasma-treated liquid, plasma medicine, reactive oxygen species, Rilac, ROS

## Abstract

**Simple Summary:**

Most cancer types of cancer can spread in the body, a process called metastasis. One particular location to which cancer cells can metastasize is the belly, also referred to as peritoneal cavity. In the peritoneal cavity, several hundred small tumors can form, making surgical removal challenging. As a complementary therapy, clinicians flush the peritoneal cavity with liquid containing chemotherapeutics. While many patients benefit from this approach, there is a lack of efficacy in some of them. To this end, we here explored the anticancer effects of a liquid enriched with free radicals as potential addition or alternative to chemotherapeutics in case of their failure. This liquid, called Ringer’s lactate, is approved as medical product and frequently employed in the clinics. We also identified a unique chemistry of components in this liquid after introduction of free radical species using a process based on gas plasma technology.

**Abstract:**

Survival rates among patients with pancreatic cancer, the most lethal gastrointestinal cancer, have not improved compared to other malignancies. Early tumor dissemination and a supportive, cancer-promoting tumor microenvironment (TME) limit therapeutic options and consequently impede tumor remission, outlining an acute need for effective treatments. Gas plasma-oxidized liquid treatment showed promising preclinical results in other gastrointestinal and gynecological tumors by targeting the tumor redox state. Here, carrier solutions are enriched with reactive oxygen (ROS) and nitrogen (RNS) species that can cause oxidative distress in tumor cells, leading to a broad range of anti-tumor effects. Unfortunately, clinical relevance is often limited, as many studies have forgone the use of medical-grade solutions. This study investigated the efficacy of gas plasma-oxidized Ringer’s lactate (oxRilac), a physiological solution often used in clinical practice, on two pancreatic cancer cell lines to induce tumor toxicity and provoke immunogenicity. Tumor toxicity of the oxRilac solutions was further confirmed in three-dimensional tumor spheroids monitored over 72 h and in ovo using stereomicroscope imaging of excised GFP-expressing tumors. We demonstrated that cell death signaling was induced in a dose-dependent fashion in both cell lines and was paralleled by the increased surface expression of key markers of immunogenic cell death (ICD). Nuclear magnetic resonance (NMR) spectroscopy analysis suggested putative reaction pathways that may cause the non-ROS related effects. In summary, our study suggests gas plasma-deposited ROS in clinically relevant liquids as an additive option for treating pancreatic cancers via immune-stimulating and cytotoxic effects.

## 1. Introduction

With more than 460,000 deaths in 2020, pancreatic cancer ranks among the leading causes of cancer mortality worldwide. Aggressive growth, a tumor-promoting microenvironment, and early metastatic dissemination in the abdominal cavity impede curative treatment options. Late diagnosis also contributes to high mortality, because patients often lack specific symptoms in the initial phase of tumor progression, thus delaying the recognition of tumor lesions [1]. Despite intensive treatment regimens that combine cytoreductive surgery and systemic chemotherapy, tumor recurrence is frequent due to high rates of incomplete excisions and poor local drug distribution [2,3,4]. Recently, an increase in disease-free survival and short-term disease control has been reported with intraperitoneal chemotherapy (IPC) for metastasized pancreatic cancer, as the direct exposure of cancer cells in the abdominal cavity improves drug efficacy [5,6]. There is also some evidence of the conversion of inoperable tumors to resectable ones after treatment with IPC. However, many tumor entities acquire chemoresistance over time, and patients experience severe drug-related side effects [7]. The combination of IPC with ROS-generating agents has produced promising results in cancer treatment [8,9]. It is argued that modulating the redox environment of cancer cells may make them more susceptible to the cytotoxic effects of IPC. We, therefore, hypothesized that combining IPC with gas plasma-based adjuvant treatment would achieve equivalent treatment efficacy at lower doses of the drugs. Translationally, this combination therapy would improve the clinical prognosis for pancreatic cancer patients with minimal drug-associated toxicity.

Due to unique biochemical alterations, tumor cells exhibit high intracellular oxidative stress, making them particularly vulnerable to lethal oxidative damage from exogenous agents [10,11]. This concept is exploited by radiation therapy, photodynamic therapy (PDT) [12], and medical gas plasma-based treatments using jets or DBDs [13]. A broad range of anti-tumor effects of solutions enriched with reactive oxygen (ROS) and nitrogen species (RNS) using this innovative technology has been described in gastrointestinal, ovarian, and pancreatic malignancies [14,15,16,17]. However, most of the investigations have used carrier solutions that are not approved for clinical application, including cell culture media, thus precluding clinical translation [18].

To address this limitation, the current study used Ringer’s lactate, a medical-grade physiological solution used clinically to replenish electrolytes, fluids, and calories. We investigated the anti-tumor efficacy of gas plasma-oxidized Ringer’s lactate (oxRilac) against two pancreatic cancer cell lines, KPC960-GFP/Luc and PDAC6606. We measured the intracellular oxidative stress imposed on the cancer cells in incubation with oxRilac. We also evaluated the direct tumor cell toxicity of this ROS- and RNS-enriched solution and the change in the immunogenicity of these often immune-resistant cancers. Key in vitro results were validated in three-dimensional tumor spheroids using high-content imaging and in GFP-expressing, vascularized tumors grown in ovo. NMR analyses and ion chromatography further indicated lactate consumption, eventually causing non-ROS-related effects. Taken together, our results underscore the potential for oxRilac solutions to be applied as adjuvant peritoneal lavage for treating pancreatic cancer.

## 2. Materials and Methods

### 2.1. Preparation of Gas Plasma-Oxidized Ringer’s Lactate

**Figure 1:**
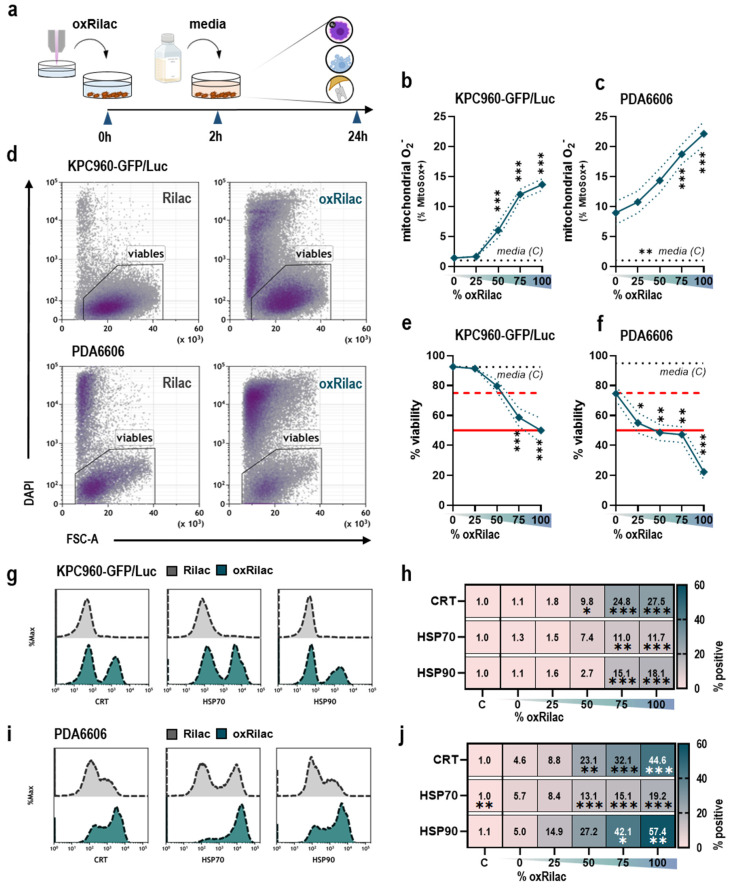
Gas plasma-oxidized Ringer’s lactate induces immunogenic cell death in pancreatic cancer cells. (**a**) A schematic overview of the in vitro experimental procedure with ROS production (purple), cell death (blue), and receptor expression analyses (schematic bubbles on the right) to indicate modes of analysis in this study; (**b**,**c**) percentage of MitoSOX Red positive (**b**) KPC960-GFP/Luc and (**c**) PDA6606 cells after exposure to serial dilutions of oxRilac solutions; (**d**–**f**) representative flow cytometry density plots (**d**) of cellular viability after oxRilac exposure and quantification thereof for (**e**) KPC960-GFP/Luc and (**f**) PDA6606 cells (IC_25_ (dashed) and IC_50_ (continuous) were indicated as red lines for each cell line); (**g**) representative flow cytometry intensity histograms of calreticulin (CRT), heat shock protein (HSP) 70, and HSP90 expression of KPC960-GFP/Luc cells; (**h**) percentage of positive cells; (**i**) representative flow cytometry intensity histograms of (CRT), HSP70 and HSP90 expression of PDA6606 cells; (**j**) percentage of positive cells. Data are mean ± standard error (SEM). Heat maps show medians. Statistical analyses were performed using two-way analyses of variance (* *p* <0.05; ** *p* < 0.01; *** *p* < 0.001).

**Figure A1:**
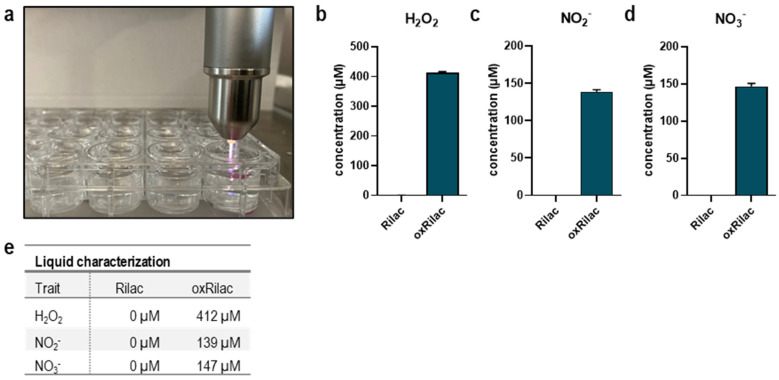
Gas plasma-generated ROS and RNS in Ringer’s solution. (**a**–**d**) kINPen conductively treating 2 mL of Ringer’s solution (**a**) for 10 min and subsequent H_2_O_2_ (**b**), NO_2_^−^ (**c**), and NO_3_^−^ (**d**) production; (**e**) summary table with final concentrations.

The plasma exposure of the Ringer’s lactate solutions was performed using the atmospheric argon plasma jet kINPen (neoplas, Greifswald, Germany). The jet was operated with argon (99.999% purity; Air Liquide, Bremen, Germany) as the feed gas at 1.5 standard liters per minute (slm). The gas was ionized at the plasma nozzle with 1 MHz, generating 1–3 W of power. Ringer’s lactate solution (Rilac) was exposed to kINPen plasma for 10 min, in 24-well plates, at a volume of 2 mL per well (Figure 1a). The treatment distance between the tip of the kINPen effluent and the liquid was 0 mm (conductive treatment), as described before [19]. Evaporation was compensated with equivalent amounts of double-distilled water immediately after treatment. The gas plasma-oxidized aliquots (oxRilac) were stored at −20 °C for all subsequent experiments. Serial dilutions (25%, 50%, 75%) of freshly thawed aliquots were prepared in Rilac before each experiment. The gas plasma oxidation of the Ringer’s lactate solutions (oxRilac) deposited 419 µM of hydrogen peroxide (H_2_O_2_), one of the mediators of in vitro anti-tumor effects of oxidized solutions (Figure A1) [20]. For experiments conducted at Drexel University, oxRilac was prepared in Germany and shipped on dry ice.

### 2.2. Detection of Gas Plasma-Derived Reactive Species

The deposition of gas plasma-derived reactive species was quantified using colorimetric assays. To determine hydrogen peroxide (H_2_O_2_) production, the *Amplex Ultra Red* assay (Thermo Fisher Scientific, Dreieich, Germany) was performed according to the manufacturer’s instructions. Fluorescence was measured at λ_ex_ 560 nm and λ_em_ 590 nm using a microplate reader (F200; Tecan, Männedorf, Switzerland). Nitrite (NO_2_^−^) and nitrate (NO_3_^−^) production were quantified using the *Griess* Assay (Biomol, Hamburg, Germany) according to the manufacturer’s protocol. Absorbance was measured at 540 nm using a multimode plate reader (M200; Tecan). Absolute concentrations in each assay were calculated against a standard curve.

### 2.3. Cell Culture and oxRilac Treatment

The murine pancreatic cancer cell line, KPC960-GFP/Luc, was cultured in Dulbecco’s Modified Eagle’s Medium (DMEM), and PDA6606 cells were grown in Roswell Park Memorial Institute (RPMI 1640; both Pan Biotec, Aidenbach, Germany) medium. Both media were supplemented with 10% fetal bovine serum, 1% glutamine, and 1% penicillin-streptomycin (all Sigma-Aldrich, Taufkirchen, Germany). Cells were incubated under standard culture conditions at 37 °C, 95% humidity, and 5% CO_2_ in a cell culture incubator (Binder, Tuttlingen, Germany). Twenty-four hours prior to the experiments, cells were seeded at a density of 1 × 10^6^ cells per well in 24-well, flat-bottom plates. Serial dilutions of oxRilac (25%, 50%, 75%) were prepared in untreated Rilac immediately before treatment. Cell culture media were removed entirely on the day of experimentation and replaced with the respective liquids. After 2 h of incubation, the liquids were removed and replaced with fresh cell culture media.

### 2.4. Flow Cytometry

Cells were analyzed for mitochondrial superoxide and the surface expression of immunogenic cell death markers twenty-four hours after exposure to oxRilac solutions using flow cytometry. For the analysis of immunogenic surface markers, cells were stained with antibodies (conjugate) targeted against calreticulin (APC-Cy7; Novus Biologicals, Wiesbaden, Germany), heat shock protein (HSP) 70 (PE/Texas Red; Santa Cruz, Heidelberg, Germany), and HSP90 (APC; Thermo Fisher Scientific, Dreieich, Germany) for 30 min at 37 °C. In addition, cells were stained with 4′,6-diamidino-2-phenylindole (DAPI; BioLegend, Amsterdam, The Netherlands) for live–dead cell discrimination and MitoSOX (Thermo Fisher Scientific) for the detection of mitochondrial superoxide. The cells were washed and analyzed using a flow cytometer (LSR Fortessa; BD Biosciences, Heidelberg, Germany). The gating and quantification of the mean fluorescence intensities were performed using *FlowJo* analysis software (BD Biosciences).

### 2.5. 3D Tumor Spheroids

To form tumor spheroids, 5 × 10^6^ cells in 100 µL of DMEM per well were seeded in a 96-well, ultra-low attachment plate (PerkinElmer, Hamburg, Germany). Cells were centrifuged at 1000× *g* for 10 min and incubated for 24 h at 37 °C, 95% humidity, and 5% CO_2_. For exposure to gas plasma-oxidized Rilac, 50 µL of media was removed and replaced with the respective solutions. After 2 h of incubation, 50 µL of DMEM was added to each well. Spheroid growth and cytotoxicity were monitored using high-content imaging (Operetta CLS; PerkinElmer) and flow cytometric analysis (CytoFLEX LX; Beckman-Coulter, Krefeld, Germany). For the quantification of cell death via high-content imaging, cells were stained with 1 µM of Sytox orange (Thermo Fisher Scientific). Image acquisition was performed at 0 h, 24 h, 48 h, and 72 h after the addition of oxRilac into the brightfield and fluorescence channels (λ_ex_ 580 nm and λ_em_ 600 nm) using a 5× air objective (NA = 0.16). The experimental setup and image analyses were performed using the analysis software *Harmony 4.9* (PerkinElmer). For flow cytometric analysis of the expression of surface markers of immunogenic cell death (ICD), spheroids were digested 24 h after treatment using accutase (BioLegend). Single-cell suspensions were stained with antibodies (conjugate) targeting HSP70 (AF647; Santa Cruz), HSP90 (PE; Enzo Life Sciences, Lörrach, Germany), CRT (Alexa Fluor 405; BioTechne, Wiesbaden, Germany), and Annexin V (PerCp-Cy5.5; BioLegend). Additionally, cells were stained with iFluor maleimide 860 (Biomol, Hamburg, Germany) for live–dead cell discrimination.

### 2.6. TUM-CAM Model

The tumor chorion-allantois model (TUM-CAM) for KPC960-GFP/Luc was established as described previously [21]. Briefly, pathogen-free, fertilized chicken eggs (Valo BioMedia, Osterholz-Scharmbeck, Germany) were incubated at 37 °C and 60% humidity in a dedicated breeding incubator (Hemel, Verl, Germany) for six days before the pointed pole was carefully punctured. On day seven, an air cell between the egg membrane and shell was created using a cannula (20G; Braun, Melsungen, Germany) for tumor cell inoculation. A silicon ring, seeded with 1 × 10^6^ KPC960-GFP-Luc in 15 µL of matrigel (Corning, Kaiserslautern, Germany), was placed on the CAM. Eggs were covered with an air-conductive dressing (Tegaderm; 3M, Neuss, Germany) and further incubated for three days to allow tumor growth, at which point, 100 µL of respective oxRilac treatment solutions were applied (day 10). On day 14, tumors were carefully excised and weighed. The GFP expression of the cells in explanted tumors was assessed using a stereomicroscope (S9; Leica, Wetzlar, Germany). The experimental setup and quantification of GFP expression were completed with LasX software (Leica). Subsequently, solid tumors were digested using a specified tumor dissociation enzyme mix and octaMACS technology (Miltenyi Biotec, Bergisch-Gladbach, Germany). Single-cell suspensions were stained with antibodies (conjugate) targeting HSP70 (Alexa Fluor 594; Santa Cruz), HSP90 (PE; Enzo Life Sciences), CRT (Alexa Fluor 647; BioTechne), Galectin-9 (PerCp-Cy5.5; BioLegend), CD324 (AF700; BioLegend), and MHC-I (Brilliant Ultraviolet 661; BD Biosciences). For live–dead cell discrimination, cells were additionally stained with iFluor maleimide 860 (Biomol). After washing, flow cytometric analyses were performed (CytoFLEX LX; Beckman-Coulter), and data were analyzed using Kaluza 2.1.3 software (Beckman-Coulter). The gating strategy to identify KPC960-GFP/Luc tumor cells in single-cell suspension first comprised the rough exclusion of cellular debris. After the exclusion of terminally dead cells (iFluor^+^), we selected cells expressing murine MHCI and GFP. Last, the gating strategy was refined based on size (FSC-A) and granularity (SSC-A).

### 2.7. Ion Chromatography

Ion chromatography was performed using the ICS-6000 Capillary System (Thermo Fisher Scientific). Separation was achieved using an AS-18 microbore column (250 × 2 mm) and a corresponding pre-column. A software-controlled eluent generator created a potassium hydroxide gradient starting at 10 mM and elevating to 20 mM in 20 min. Detection was achieved after hydroxide ion suppression using an amperometric conductivity sensor and UV detector (206 nm). Oxidized (ox) Rilac samples were exposed to gas plasma for 1 min and 3 min and diluted at a rate of 1:10 or 1:100 before injecting a 5 µL aliquot of each sample. Peak identification was performed through a comparison with the retention time of standards (Thermo Fisher Scientific) and quantified using calibration curves. Data handling was done with Chromeleon 7.3 software (Thermo Fisher Scientific).

### 2.8. Nuclear Magnetic Resonance (NMR) Spectroscopy

^1^H-nuclear magnetic resonance (^1^H-NMR) spectroscopy was performed 5 h after gas plasma treatment, as described previously [22]. In brief, 400 µL of the sample was mixed with 200 µL of sodium hydrogen phosphate buffer (0.2 mol/L; pH 7.0), made up of 50% D_2_O, including 1 mmol/L of 3-trimethylsilyl-[2,2,3,3-D4]-1-propanoic acid, for ^1^H-NMR analysis. An AVANCE-II 600 NMR spectrometer (Bruker, Bremen, Germany) was operated by TOPSPIN 3.2 software. Qualitative and quantitative data analyses were conducted using TOPSPIN 4.0.9 (Bruker Biospin) and Chenomx NMR Suite v9 (Chenomx, Edmonton/Alberta, Canada).

### 2.9. Statistical Analysis

Graphing and statistical analyses were completed using Prism 9.5.0 (GraphPad Software, San Diego, CA, USA). Statistical significance was established using either *t*-tests or analyses of variance (ANOVAs), as appropriate. Data show the mean ± the standard error of the mean (SEM).

## 3. Results

### 3.1. Gas Plasma-Oxidized Ringer’s Lactate Induces Immunogenic Cell Death in Pancreatic Cancer Cells

Gas plasma-oxidized liquids are enriched with ROS and RNS, which are known to produce a broad range of oxidation-induced, anti-cancer effects in various tumor entities. The present study characterized the tumor toxicity and immunogenicity of gas plasma-oxidized Ringer’s lactate (Rilac), a solution routinely used in clinical procedures, in a model of pancreatic cancer (Figure 1a). A dose-dependent increase in mitochondrial superoxide was observed twenty-four hours after exposure to serial dilutions of oxRilac in KPC960-GFP/Luc (Figure 1b) and PDA6606 (Figure 1c) cells, indicating the induction of oxidative stress in both cell lines. This increase in oxidative stress can lead to lethal damage in cellular macromolecules, resulting in cell death. An analysis of cellular viability (Figure 1d) confirmed a dose-dependent decrease after twenty-four hours in both KPC960-GFP/Luc (Figure 1e) and PDA6606 cells (Figure 1f). Of note, PDA6606 cells had higher basal superoxide levels, as seen in the cells incubated in untreated Rilac solutions. This was reflected in the higher tumor toxicity of PDA6606 cells compared to KPC960-GFP/Luc cells.

The most promising anti-cancer therapeutic strategies engage the immune system. As tumor cells acquire distinct mechanisms to evade the immune system, one approach is to enhance the immunogenicity of cancer cells. To explore whether oxRilac triggers these responses, the appearance of pro-immunogenic surface markers on dying cancer cells was evaluated. After exposure to oxRilac solutions, the surface translocation of key molecules involved in immunogenic cell death (ICD), such as calreticulin (CRT) and heat-shock proteins (HSP) 70 and 90 (Figure 1g,i), was significantly increased in KPC960-GFP/Luc (Figure 1g) and PDA6606 cells (Figure 1i).

### 3.2. Gas Plasma-Oxidized Ringer’s Lactate Induces Tumor Toxicity in Pancreatic Cancer Spheroids

**Figure 2:**
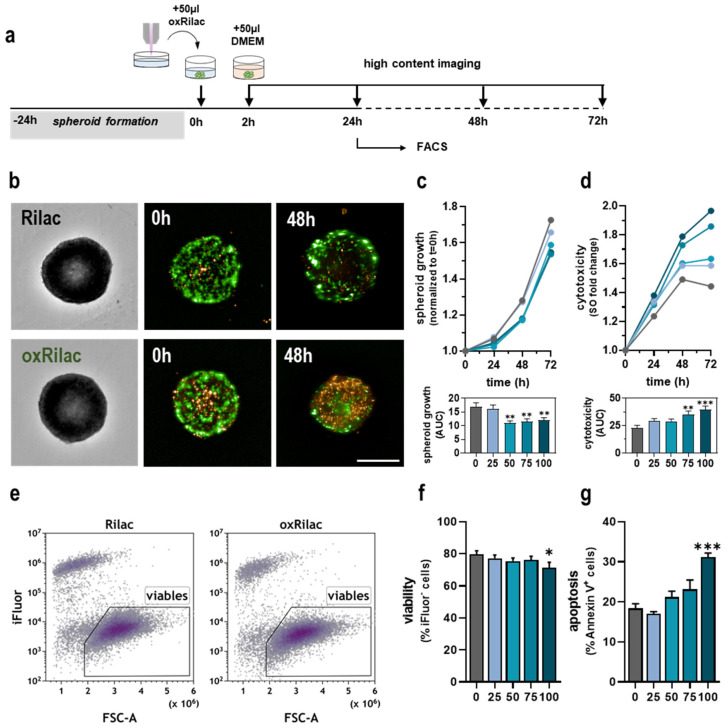
Gas plasma-oxidized Ringer’s lactate induces cytotoxicity in pancreatic cancer spheroids. (**a**) Schematic overview of the spheroid treatment procedure; (**b**) representative images of KPC960-GFP/Luc spheroids at 0 h and 48 h after treatment in brightfield, GFP, and Sytox Orange (SO) fluorescence channels; (**c**) spheroid growth over 72 h after treatment and quantification of the area under the curve (AUC); (**d**) cytotoxicity over 72 h after treatment with oxRilac and quantification of the AUC; (**e**) representative flow cytometry density plots of viable cells 24 h after exposure to oxRilac and (**f**) quantification thereof; (**g**) quantification of Annexin V^+^ cells for detection of apoptotic cells. Data are mean ± standard error (SEM). Statistical analysis was performed using two-way analyses of variance (* *p* < 0.05; ** *p* < 0.01; *** *p* < 0.001). Scale bar = 500 µm.

Three-dimensional, extracellular matrix-producing tumor spheres allow for the advanced screening of treatment strategies in vitro. Therefore, we validated the tumor toxicity of oxRilac solutions, observed in the monolayer cell cultures presented above, in pancreatic cancer spheroids (Figure 2a). The monitoring of spheroids, using high-content imaging for 72 h after exposure to oxRilac solutions (Figure 2b), revealed a significantly lower growth rate that was not dose-dependent (Figure 2c). Growth kinetics can be altered due to a temporary cell cycle delay or exposure to a cytotoxic agent. To determine which of the two mechanisms contributed to the lower growth rate of spheroids, KPC960-GFP/Luc cells were stained with Sytox orange to label terminally dead cells after treatment. Exposure to serial dilutions of oxRilac solutions confirmed a dose-dependent, tumor-toxic effect on KPC960-GFP/Luc cells (Figure 2d). A flow cytometric analysis of digested tumor spheroids was performed after 24 h to elucidate the mode of cell death and distinguish necrotic from apoptotic tumor cells (Figure 2e). Strikingly, only a slight decrease in cellular viability was observed after 24 h, which was in line with high-content imaging time-lapse experiments (Figure 2f). A hallmark of cells undergoing apoptotic cell death is the translocation of the phospholipid phosphatidylserine to the outer cell membrane, which is detected using Annexin V staining. Our results showed increased levels of phosphatidylserine in KPC960-GFP/Luc cells in spheroids after exposure to a 100% oxRilac solution, indicating the induction of pro-apoptotic signaling pathways (Figure 2g).

### 3.3. Gas Plasma-Oxidized Ringer’s Lactate Reduces Tumor Burden in Ovo in Conjunction with Increased Display of Immunogenic Surface Markers

**Figure 3:**
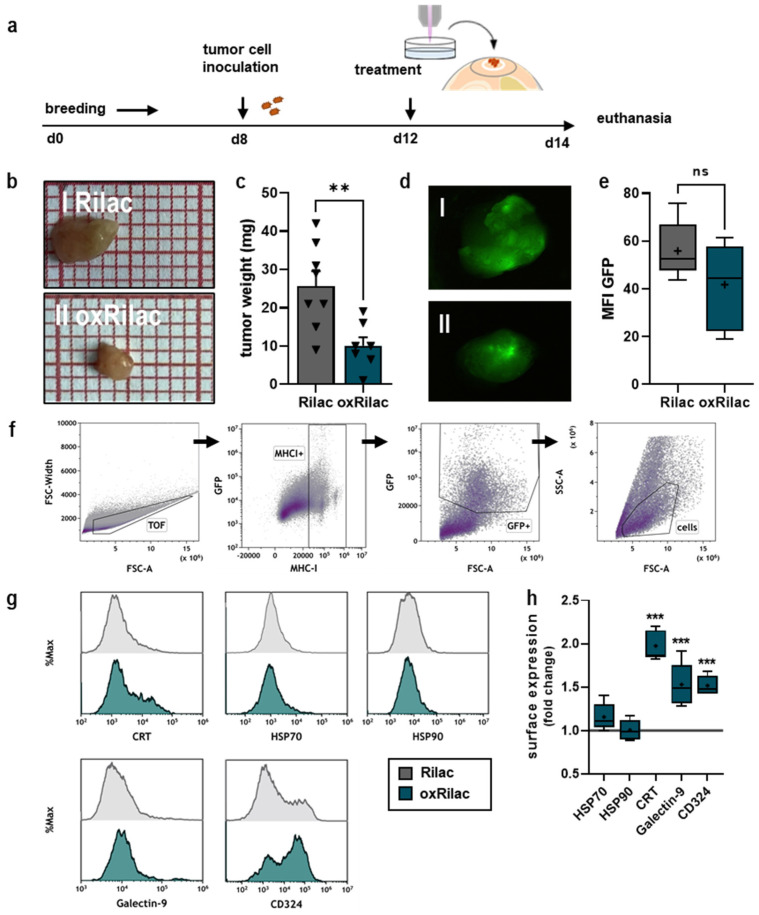
Gas plasma-oxidized Ringer’s lactate reduces tumor burden in ovo paralleled by the upregulation of immunogenic surface markers. (**a**) Schematic overview of the treatment procedure in ovo; (**b**) representative images of explanted tumors after treatment with (**b**(**I**)) Rilac and (**b**(**II**)) oxRilac; (**c**) tumor weights; (**d**) representative fluorescent images of GFP expression in explanted (**d**(**I**)) Rilac-treated and (**d**(**II**)) oxRilac-treated tumors; (**e**) GFP quantification; (**f**) representative flow cytometry density plots of the gating strategy for single-cell analysis of digested in ovo tumors; (**g**) representative flow cytometry intensity histograms of HSP70, HSP90, CRT, Galectin-9, and CD324 expression; (**h**) FACS quantification. Data are mean ± standard error (SEM). Boxplots show minimum to maximum values, with the mean indicated as (+). Statistical analyses were performed using *t*-tests or two-way analyses of variance (** *p* < 0.01 and *** *p* < 0.001).

As a semi-in vivo model, the tumor-chorioallantoic membrane assay (TUM-CAM) obeys the 3R’s—reduce, replace, refine—and no ethical approval is needed because the experiments are terminated by day fourteen of ontogenesis. The TUM-CAM model of KPC960-GFP/Luc cells was used to investigate the anti-tumor efficacy of a 100% oxRilac solution on solid, neovascularized tumors (Figure 3a). Tumors were excised on day fourteen and compared for growth (Figure 3b). An assessment of tumor weight revealed a significant reduction in tumor burden after exposure to oxRilac solutions compared to untreated controls (Figure 3c). Tumor weight in vivo is an integration of proliferating tumor cells and the stroma components. Therefore, using weight as the sole surrogate marker for the anti-tumor efficacy of therapy can be misleading. We determined the number of proliferating tumor cells in ovo-grown tumors by quantifying GFP fluorescence intensities using stereomicroscope imaging (Figure 3d). A reduction in GFP^+^ tumor cells was detected after exposure to oxRilac solutions (Figure 3e), indicating direct effects on tumor cells. Explanted tumors were digested and analyzed via flow cytometry to investigate the surface marker expression of single-cell suspensions (Figure 3f,g). After exposure to oxRilac solutions, a significant increase in the surface display of CRT, Galectin-9, and CD324, but not HSP70 or HSP90, was observed (Figure 3h).

### 3.4. Gas Plasma Oxidation of Ringer’s Lactate Solution Leads to a Limited Organic Component Breakdown 

**Figure 4:**
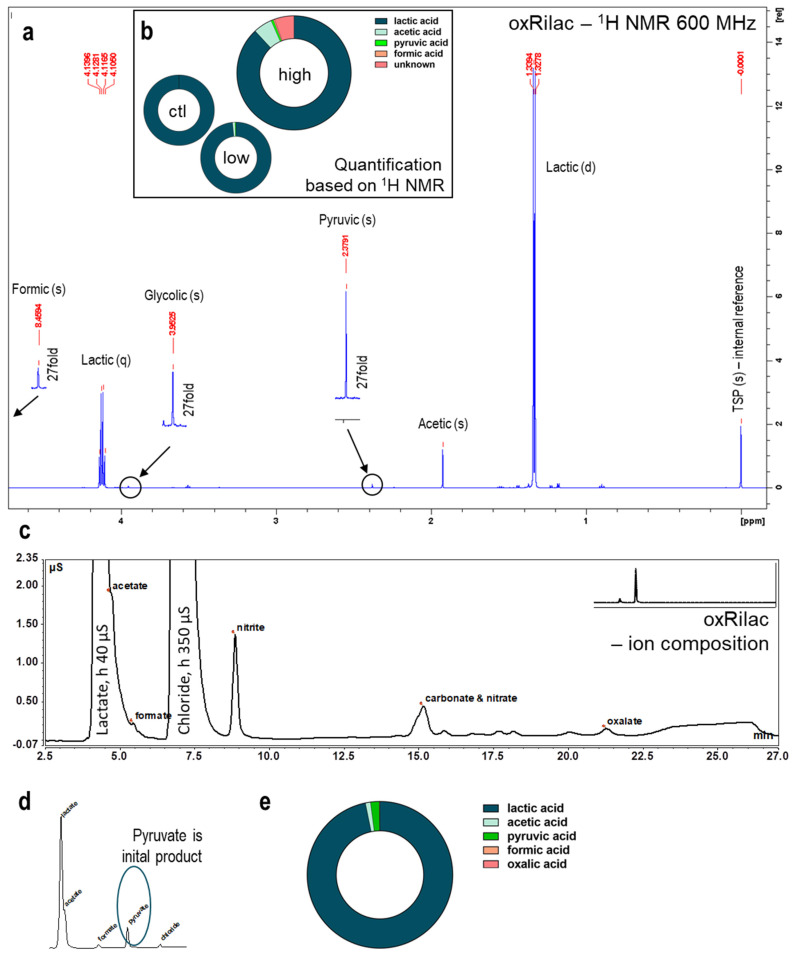
Gas plasma treatment of Ringer’s lactate solutions yields lactate oxidation. (**a**) ^1^H-NMR spectrum of oxRilac in the range from δ = 0 to δ = 4.8 reveals lactate signals (doublet δ = 1.33, quadruplet δ = 4.12), acetic acid as major product (singulet δ = 1.91), smaller signals belonging to pyruvic acid (singulet δ = 2.38) and glycolic acid (singulet δ = 3.95), and formic acid (singulet δ = 8.46), apart from contaminants (disinfectants) on the control level; (**b**) quantification of educts and products in control (Ringer’s lactate solution with 25 mM of lactate) and extended (10 min) gas plasma-treated samples (22.1 ± 0.5 mM of lactate, 1.5 ± 0.1 mM of known products, 1.4 ± 0.1 mM of unknown products) as well as short treatment (1 min) conditions (1.5% products, mainly acetic acid); (**c**) oxRilac ion chromatogram showing lactate (R_T_ = 4.13 min) and chloride ions (R_T_ = 6.55 min); acetate (lactate peak shoulder), formate, and oxalate; (**d**) pyruvate is detectable through ion chromatography immediately after gas plasma treatment in Ringer’s solution without chloride ions; (**e**) ion chromatography quantification reveals abundant pyruvate (≈430 µM) and acetate (≈250 µM) and only minor traces of oxalate (25 µM) and formate (6 µM).

**Figure 5:**
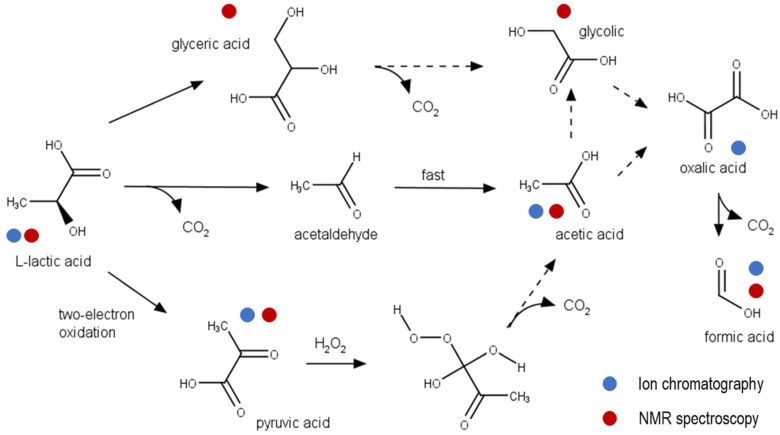
Reaction schematic of gas plasma oxidation of Ringer’s solution’s main constituent, lactate, reveals acetate and carbon dioxide as major products. The decarboxylation of lactate yields acetaldehyde that is immediately oxidized to acetic acid. In parallel, one-electron oxidations yield pyruvic acid, which reacts with deposited H_2_O_2_, yielding acetic acid again. Minor side reactions via glycerate and glycolic acid occurred. Terminal oxidation via oxalate and formic acid yield carbon dioxide as the ultimate product.

The anti-tumor effects of gas plasma-oxidized solutions have been predominantly linked to long-lived ROS/RNS—H_2_O_2_, in particular. However, the oxidation of carrier solutions supplemented with organic compounds might cause their breakdown and the induction of additional non-ROS effects specific to the composition of the solution that is being investigated. Rilac contains significant amounts of sodium lactate, which is utilized in primary cellular metabolism via lactate dehydrogenase-driven oxidation to produce pyruvic acid and a subsequent introduction into the TCA cycle. Depending on the treatment time, a maximum of 10% of the lactate was oxidized by the gas plasma-derived species to yield pyruvate and acetaldehyde as initial products (Figure 4 and Figure 5). Structural analyses, using nuclear magnetic resonance spectroscopy (^1^H-NMR), revealed fingerprint spectra were signals of unmodified lactate dominate (quadruplet at δ = 4.12 ppm and doublet at δ = 1.33 ppm), indicating that only minor amounts of the original 25 mM were turned over (Figure 4a). Extended gas plasma treatment (10 min) yielded 3 mM of products, of which, 1.5 mM were known and 1.4 mM were unknown products (Figure 4b). Given the signature profiles, the latter might be carbon dioxide and/or carbonate ions. The lactate signals also dominated in ion chromatography, with lactate (R_T_ = 4.13 min) and chloride (R_T_ = 6.55 min) being abundantly present in the chromatogram (Figure 4c). Several downstream reactions occurred, leading to the final product: carbon dioxide. Depending on the treatment and the storage time after treatment, a mix of intermediate products was observed. Among these, were the early and unstable pyruvate and the late (and stable) acetate dominate. Acetate was also formed by the direct decarboxylation of lactate (Figure 5), yielding acetaldehyde that was immediately further oxidized into the corresponding acid. Both photocatalytic and one-oxygen oxidizers, such as OH radicals, can contribute to this reaction [23]. In ^1^H-NMR analyses, no traces of any aldehyde hydrogen atoms (δ ≥ 9 ppm) were detected, ruling out potentially toxic byproducts. In this range of chemical shifts, only the hydrogen of formate (δ = 8.46 ppm) was found. Aside from this, small amounts of glycolic acid, glyceric acid, formic, and oxalic acid were observed in the ion chromatography and NMR analyses (Figure 4a,c). Overall, the lactate oxidation products’ concentration amounted to a maximum of 5–7% of the initial lactate and was, ultimately, too low to substantially impact activity. It may be conjectured that the formation of carbon dioxide, due to the decay of lactate, contributes to the formation of peroxynitrite-equivalent peroxycarbonates [24]. 

## 4. Discussion

Developing innovative therapeutic strategies combined with highly specialized surgical interventions has greatly improved patients’ outcomes for many malignancies. However, in patients with certain gastrointestinal tumors and pancreatic cancer, in particular, early tumor dissemination, a high rate of surgical R1 resections [3,25], and a cancer-supportive tumor microenvironment (TME) [26] impede the chance of tumor remission at early stages. Mounting evidence suggests that the intraperitoneal administration of gas plasma-oxidized liquids, as part of a multi-regimen therapeutic strategy, could benefit patients suffering from these hard-to-treat tumors [27,28,29]. However, many of these preclinical studies forewent medical-grade carrier solutions, thus limiting their translational potential [14,29,30]. The present study addresses this gap using Ringer’s lactate, commonly applied in clinical practice. Oxidized Ringer’s lactate solution elicited profound cytotoxic and immunogenic activity in monolayer, spheroid, and in ovo experiments. Functional experiments were complemented by ^1^H-NMR analyses and ion chromatography of oxRilac solutions to evaluate lactate consumption linked to non-ROS related effects, as reported recently [31,32]. These promising outcomes will pave the path for using such treatment modalities in future clinical investigations.

Upon excessive oxidative stress, cellular stress response mechanisms potentially fail to preserve irreversible damage to cellular macromolecules, and the induction of cell death signaling occurs. Beneficial anti-tumor effects of gas plasma-oxidized liquids have been shown in pre-clinical models of glioblastoma [33], lung [34], colorectal [15,35], ovarian [27,36], and also pancreatic cancers [30,37]. Gas plasma-oxidized liquids are suggested to target the tumor redox state, predominantly via H_2_O_2_ deriving from secondary reactions of gas-phase species in the carrier liquid [38]. In oxRilac solutions, gas plasma oxidation yielded in the deposition of 419 µM of H_2_O_2_, which was sufficient to oxidize both KPC960-GFP/Luc and PDA6606 cells at all tested dilutions of the oxidized liquid. Cellular oxidation was paralleled by the induction of cell death in both cell lines, although a higher sensitivity of PDA6606 cells was observed. The cytotoxic effects of untreated Rilac solutions on PDA6606 cells may be attributed to media starvation during the 2 h exposure period. Moreover, oxRilac solutions confirmed their potential to reduce tumor burden in three-dimensional pancreatic tumor spheroids and in ovo. High-content imaging was combined with flow cytometric analyses to specify the mode of cell death in KPC960-GFP/Luc spheroids. One hallmark of apoptotic cell death is the translocation of phosphatidylserine to the outer part of the gas plasma membrane [39], which was observed after exposure to oxRilac solutions. Several studies suggest that oxidation-induced cell death signaling, upon exposure to gas plasma-oxidized liquids, mainly accounts for pro-apoptotic processes. In this regard, during the activation of enzymes involved in pro-apoptotic signaling cascades, e.g., caspase 3/7 [40], an increase in pro-apoptotic Bax over Bcl-2 [41] and the mitochondrial release of cytochrome c [42] have been reported.

While apoptosis is often described as ‘silent’ cell death, it can still be immunogenic [43]. Strengthening the body’s immune defense extends standard cytostatic treatment regimes with a powerful tool that has impressively changed the prognosis of severe diseases [44]. As cancer cells develop distinct mechanisms to evade recognition by immune cells, one approach is to trigger the release and surface display of pro-immunogenic damage-associated molecular patterns (DAMPS), e.g., ATP, CRT, and HSPs, on dying cancer cells [45,46]. CRT can act as an ‘eat me’ signal for innate immune cells (dendritic cells) upon binding to its receptor density lipoprotein-related receptor-1 (LRP-1, CD91) [47]. This eventually results in their subsequent maturation and enhances the phagocytosis of tumor material [48,49]. Activated dendritic cells may migrate into draining lymph nodes, where they present processed tumor antigens to T cells to initiate an anti-tumor immune response [50,51]. A significant upregulation of pro-immunogenic surface markers, including CRT, was achieved in KPC960-GFP/Luc and PDA6606 cells in vitro, which is in line with previous observations [30,38,52]. However, an increase in the expression of Galectin-9 in ovo was observed. This molecule is known to expand regulatory T cells and contribute to T cell exhaustion [53]. Nonetheless, it was shown by Van Loenhout and colleagues that gas plasma-oxidized PBS targets not only pancreatic cancer cells but also immunosuppressive pancreatic stellate cells. By contrast, an increase in activated dendritic cells was seen in vitro [52]. The increase in myeloid infiltrates in murine pancreatic cancer tumors after the intraperitoneal lavage of oxidizing medium highlights the immuno-modulating effects noted in our study. The gas plasma-oxidized medium could also reverse the immunosuppressive tumor microenvironment in pancreatic cancer lesions [54]. Together, these results [54] underline the potential of gas plasma-oxidized liquids to serve as a pro-immunogenic anti-cancer agent. In addition to surface markers related to immunoregulatory processes, the upregulation of the adhesion protein E-cadherin (CD324) was found. The downregulation of adhesion markers upon an epithelial–mesenchymal transition (EMT) is essential to the dedifferentiation processes of aggressively growing tumor cells and increases the risk of metastasis. Previous risk assessments of direct gas plasma treatment on pancreatic cancer cells [55,56] and melanoma cells [57] have already indicated that EMT markers are not increased after gas plasma treatment, which is in line with our findings.

Long-lived ROS have a prominent role in the anti-tumor effects elicited by gas plasma-oxidized solutions. Nevertheless, synergistic non-ROS-related effects may play a role in solutions containing organic compounds. Tanaka and colleagues were the first to outline the presence of lactate oxidation products as important additional tumor toxic agents in oxRilac solutions [31]. From a chemical viewpoint, the oxidation of lactate as the major organic constituent of Rilac solution during gas plasma treatment yields the formation of small organic acids at the expense of short-lived reactive species. With the chosen approach, prolonged exposure of Rilac solution to gas plasma consumed only a small fraction, never exceeding 10% of the lactate initially present. This is contrasted by results presented by Tanaka and colleagues, who observed more substantial consumption (12% in 5 min) [32]. The formed breakdown products were identical (formate, pyruvate, acetate), except for glyoxylate and 2,3-dimethyl tartrate, which were not detected (glyoxylate) or detected only in trace amounts (2,3-dimethyl tartrate) in our study. Despite similar feed gas composition, the gas plasma source used by Tanaka has a different electrode design and was optimized to generate a large amount of atomic oxygen [58]. It is reasonable to assume that differences in the subsequent ROS/RNS chemistry profiles, further substantialized by higher levels of long-lived nitrogen species in the case of the kINPen, justify the observed differences. Further, ozone production by the kINPen [59] could have contributed to these findings. While 2,3-dimethyl tartrate showed profound cytotoxic effects, the acids determined in the present study are non-toxic and part of primary or secondary cell metabolism. Since the concentrations remain low to moderate, no direct effect of these acids can be expected. The chelating agent, oxalic acid, which was found in small amounts (up to 30 µM), may drive oxidative stress by modulating iron ion transport. The initially generated pyruvic acid is not stable in pro-oxidant conditions, yielding to the consumption of equimolar amounts of hydrogen peroxide after gas plasma exposure. The rate constant is low (2.360 ± 0.198 M^−1^ s^−1^, [60]), suggesting that the activity of the solution decreases with time [14]. However, once the pyruvate is consumed, this effect comes to a complete end. Taken together, the present study highlights the potential of gas plasma-oxidized Ringer’s lactate to be applied in intraperitoneal lavage to treat patients suffering from advanced pancreatic cancer.

## Data Availability

The underlying data of this manuscript can be retrieved from the corresponding author upon reasonable request.
